# A High-Throughput Microfluidic Cell Sorter Using a Three-Dimensional Coupled Hydrodynamic-Dielectrophoretic Pre-Focusing Module

**DOI:** 10.3390/mi14101813

**Published:** 2023-09-22

**Authors:** Mohammad Aghaamoo, Braulio Cardenas-Benitez, Abraham P. Lee

**Affiliations:** 1 Department of Biomedical Engineering, University of California Irvine, Irvine, CA 92697, USA; maghaamo@uci.edu (M.A.); braulio.cardenas@uci.edu (B.C.-B.); 2 Center for Advanced Design & Manufacturing of Integrated Microfluidics (CADMIM), University of California Irvine, Irvine, CA 92697, USA; 3 Department of Mechanical & Aerospace Engineering, University of California Irvine, Irvine, CA 92697, USA

**Keywords:** microfluidics, dielectrophoresis, high-throughput cell sorting, hydrodynamic-dielectrophoretic 3D cell pre-focusing, theoretical and numerical modeling

## Abstract

Dielectrophoresis (DEP) is a powerful tool for label-free sorting of cells, even those with subtle differences in morphological and dielectric properties. Nevertheless, a major limitation is that most existing DEP techniques can efficiently sort cells only at low throughputs (<1 mL h^−1^). Here, we demonstrate that the integration of a three-dimensional (3D) coupled hydrodynamic-DEP cell pre-focusing module upstream of the main DEP sorting region enables cell sorting with a 10-fold increase in throughput compared to conventional DEP approaches. To better understand the key principles and requirements for high-throughput cell separation, we present a comprehensive theoretical model to study the scaling of hydrodynamic and electrostatic forces on cells at high flow rate regimes. Based on the model, we show that the critical cell-to-electrode distance needs to be ≤10 µm for efficient cell sorting in our proposed microfluidic platform, especially at flow rates ≥ 1 mL h^−1^. Based on those findings, a computational fluid dynamics model and particle tracking analysis were developed to find optimum operation parameters (e.g., flow rate ratios and electric fields) of the coupled hydrodynamic-DEP 3D focusing module. Using these optimum parameters, we experimentally demonstrate live/dead K562 cell sorting at rates as high as 10 mL h^−1^ (>150,000 cells min^−1^) with 90% separation purity, 85% cell recovery, and no negative impact on cell viability.

## 1. Introduction

Isolating and sorting key cell types from heterogenous populations is an essential step in medicine and biotechnology. Major applications include, but are not limited to, disease diagnosis and prognosis, cell therapy manufacturing, and single/rare cell analysis [1,2,3,4]. For example, the detection, isolation, and characterization of circulating tumor cells (CTCs) and their clusters play a critical role in early cancer diagnosis, monitoring disease progression, and developing personalized therapy [5,6]. Another promising application is cancer immunotherapy, such as chimeric antigen receptor (CAR) T-cell therapy, where high throughput and fine separation of immune cells is an important step in cell therapy manufacturing [4,7]. Currently, the three main isolation methods widely used in research labs and clinical settings are density gradient centrifugation (DGC), fluorescence-activated cell sorting (FACS), and magnetic-activated cell sorting (MACS). However, these techniques have limitations. Although DGC has clinically relevant isolation yield, it lacks isolation specificity, as it relies solely on physical properties such as cell density, is time-consuming, and can result in unwanted ex vivo leukocyte activation [8]. On the other hand, FACS and MACS solve the specificity issue by tagging cells with antibodies conjugated to fluorescent molecules or magnetic beads, respectively. In FACS and positive-selection MACS, permanent labeling of target cells limits their usefulness for downstream applications, whereas negative-selection approaches focused on immunodepletion of unwanted cell populations incur higher costs and increased design complexity by requiring large antibody panels [9]. In addition, at high sorting rates, isolation sensitivity reduces, leading to significant cell loss, and the high cost of instruments and reagents further adds to the disadvantages of these techniques.

To address these limitations, microfluidic technologies have emerged as alternative candidates to replace conventional cell sorting methods [1]. Notable techniques include inertial microfluidics [10,11,12], acoustic-based microfluidics [13,14,15], dielectrophoresis (DEP) [16,17], magnetophoresis [18], deterministic lateral displacement [19,20], pinched flow fractionation [21], microfiltration [22], and cellular immobilization [23,24]. Specifically, DEP is a powerful label-free approach that exploits both electrophysiological properties and the size of cells for sorting [25]. It has been shown that DEP can distinguish cells even with subtle differences in their morphological and dielectric properties [26,27,28]. As a result, DEP has been successfully used for sorting stem cells [29,30], cellular blood components [31,32,33], malignant from healthy cells [34,35,36], live from dead cells [37,38,39], etc. Despite encouraging results, one major problem is that most DEP designs suffer from a low cell sorting throughput, typically below 1 mL h^−1^, which limits their widespread use. This is especially more challenging for DEP designs based on conventional planar interdigitated array (IDA) electrodes. Although IDA electrodes are a popular choice for DEP-based systems due to their low cost, simple fabrication, and ease of integration with microfluidic modules, sorting efficiency significantly reduces at high flow rates as hydrodynamic forces dominate DEP forces (i.e., DEP forces cannot deflect and separate cells of interest). One possible solution is to change the channel dimensions to keep the flow velocity and, consequently, hydrodynamic forces low. In microfluidic channels with rectangular-shaped cross-sections (as fabricated in this work, based on soft lithography), the average fluid velocity can be expressed as follows:

(1) $$V_{ave}=\frac{Q}{\rho wH},$$

where Q is the flow rate, ρ is the fluid density, w is the channel width, and H is the channel height. Based on Equation (1), increasing channel height, width, or a combination of both keeps the flow velocity low at a given flow rate. Increasing channel width, however, necessitates an increase in the size (length) of electrode arrays. This can potentially lead to a reduction in the impedance of the electrode/electrolyte system and, consequently, the inefficacy of DEP force by affecting the electric field strength [40]. In addition, for positive DEP-based sorting, cells need to slide on top of electrodes, which can result in unwanted cell-to-electrode adhesion. On the other hand, increasing channel height is an alternative approach to reducing flow velocity at high flow rates. Nonetheless, this results in weaker DEP force on cells flowing near the top of the microchannel because the electric field exponentially decays in the direction normal to the plane for planar IDA electrodes. To address this, designs based on vertical electrodes provide an effective electric field with minimized decay across the channel height [41,42]. However, the complex fabrication of vertical electrodes is an important tradeoff to consider. 

**Figure 1:**
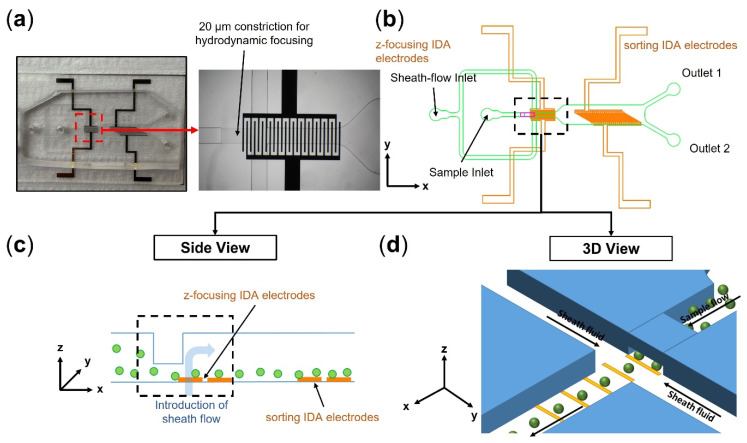
(**a**) Image of the microfluidic DEP sorter. (**b**) The 2D schematic of the DEP sorter that integrates a hydrodynamic-DEP 3D cell focusing module upstream of the main sorting region to keep cells close to the electrode, where hydrodynamic and DEP forces can still be balanced at high flow rates for efficient cell separation. (**c**) Side-view and (**d**) 3D-view schematics of hydrodynamic-DEP 3D cell focusing module.

Here, we present a microfluidic DEP chip with planar IDA electrodes for cell sorting with rates as high as 10 mL h^−1^ (>150,000 cells min^−1^). For working at high flow rate regimes, our design incorporates a coupled hydrodynamic-DEP 3D cell focusing module upstream of the main sorting region to keep cells close to the IDA electrodes, where the electric field and, consequently, DEP force are effective and can balance the hydrodynamic force. Compared to conventional DEP designs with planar IDA electrodes that are limited by low channel heights (<100 µm), such an integrated z-focusing module enables the fabrication of higher channel heights (250 µm in this study). Figure 1 shows the schematic design of our proposed DEP system. The coupled hydrodynamic-DEP 3D cell focusing module utilizes a multilayer microchannel design. Once cells are introduced into the chip, they pass through a narrow constriction (20 µm in height). Upon exiting the constriction, the sheath flow focuses the cells across both y (channel width) and z directions (channel height). By intermittently turning on the DEP z-focusing electrodes, a downward pulling force is also applied on cells via positive DEP that eliminates the need for unwanted high sheath flow rates to keep cells close to the bottom of the microchannel. 

In this study, we first developed a theoretical model to better understand the role of the z-focusing module. The theoretical model presents the expressions that describe the dependence of the total hydrodynamic and electrostatic forces on cells, as a function of their position relative to the sorting electrodes. Based on the model, we calculated the critical cell-to-electrode distance ($h_{critical}$), below which sorting can be achieved at high flow rates. To focus cells below $h_{critical}$, we adopted numerical modelling to find optimum operating parameters (i.e., flow rate ratio and electric voltage applied to z-focusing electrodes) for the coupled hydrodynamic-DEP 3D cell focusing module. The optimum parameters were then used to experimentally evaluate the sorting performance of the proposed system at low (≤0.1 mL h^−1^), moderate (≤1 mL h^−1^), and high (>1 mL h^−1^) flow rate regimes. 

## 2. Theoretical Model

### 2.1. Hydrodynamic Forces

**Figure 2:**
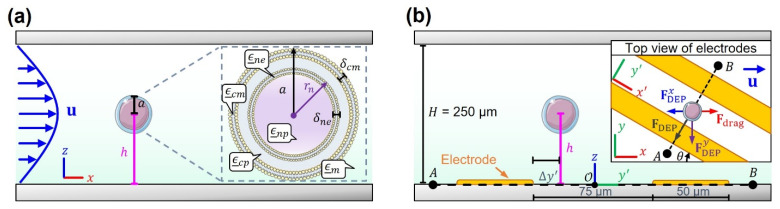
(**a**) Schematic of the hydrodynamic set-up resulting in drag forces on cells located at $\left(0,0,h\right)$. Inset: the multi-shell model used to calculate cell dielectric properties. (**b**) Schematic of cell-to-electrode positioning. Inset: top view of the electrodes and the rotated coordinate system.

In this section, we introduce the equations used to compute the total hydrodynamic forces on spherical cells flowing relative to sorting IDA electrodes in Figure 1b–d. Side-view schematics of cells as they flow past the sorting IDA electrode region are shown in Figure 2. Based on the coordinate orientations in Figure 2, the x-component of the total fluidic drag on stationary, ideally spherical cells with radius a located at a distance h from the electrodes can be found via integration of the hydrodynamic stress tensor (N) on the cell’s surface S:

(2) $$F_{\mathrm{drag}}^{x}=\oint_{S}dA\hat{n}\cdot N\cdot \hat{x},$$

where $dA\hat{n}$ is a surface element, $\hat{x}$ is the unit vector in the x-direction, and the stress tensor is defined as

(3) $$N=-pI+\eta \left[\nabla u+{\left(\nabla u\right)}^{†}\right],$$

where $u\left(x,y,z\right)$ is the vector flow field, p is the pressure scalar field, η is the dynamic viscosity of the suspension media, and I is the identity tensor. We note that Equation (2) describes the total force $F_{\mathrm{drag}}^{x}$ experienced by a spherical particle that is assumed to be held in place by another counterbalancing force (in this case, a DEP force). This condition thus allows us to calculate when DEP and hydrodynamic forces are comparable.

At the start of the sorting IDA electrode region (Figure 1b), the microchannel of the rectangular cross-section (height H, width w) can be assumed to have fully developed laminar flow as an approximation. Under these conditions and in the absence of flowing cells, u is given via a rectangular Poiseuille flow profile [43]:

(4) $$u\left(0,y,z\right)=\frac{g}{\eta }\frac{4H^{2}}{\pi^{3}}\sum_{n=1,3,5,\ldots }^{\infty }{\left(-1\right)}^{\frac{\left(n-1\right)}{2}}\frac{1}{n^{3}}\left(1-\frac{\cosh\left(n\pi y/H\right)}{\cosh\left(n\pi w/2H\right)}\right)\cos\left(n\pi \left(z-\frac{H}{2}\right)/H\right)\hat{x},$$

where the applied pressure gradient along the x-axis is given by g=Δp/L for a given flow rate Q, with Δp being the pressure drop after a distance L along x. A first approximation to calculate the $F_{\mathrm{drag}}^{x}$ as a function of h for a small spherical particle (in comparison to the scale of the flow field variations) involves using the Stokes drag equation [44] in combination with Equation (4):

(5) $$F_{\mathrm{drag}}^{x}=6\pi \eta au_{x},$$

where $u_{x}\left(h\right)=u\left(0,0,h\right)\cdot \hat{x}$. A rectangular channel with an arbitrary aspect ratio, however, in general, does not admit an analytic calculation of $F_{\mathrm{drag}}^{x}$ for arbitrary h. Therefore, we have performed a finite element analysis simulation of the flow field in the presence of a fixed (i.e., not freely rotating) spherical particle placed at r=(0,0,h) from the flat electrodes. The perturbed flow field has been used to compute the integral in Equation (2) for a parametric study of h, spanning the device bottom to the device half height (H/2), which is presumably the point with the strongest drag.

### 2.2. Dielectrophoretic Forces

In addition to the hydrodynamic forces experienced by the flowing particles, there are also electrostatic stresses that are a result of the induced polarization of the dielectric cells suspended in low-conductivity media. This polarization, the product of the frequency-dependent applied electric field Er,t=ReE¯reiωt, varies in magnitude with the vertical distance h between cells and electrodes (Figure 2b). Here, $\underline{E}\left(r\right)$ represents the phasor electric field vector containing the spatial and polarization information of the field created by the electrodes (all underlined quantities are complex). 

As the distance between cells and electrodes h decreases, the DEP holding forces on cells become stronger due to the increased polarization. A first approximation to calculating the total electrostatic force on a cell-centered at position r involves using the time-averaged DEP force [45,46]:

(6) 〈FDEPr〉=πϵma3ReK¯1∇E¯r2,

where a is the radius of the spherical cell with complex permittivity ${\underline{\epsilon }}_{p}$, immersed in medium with real permittivity component $\epsilon_{m}$, and ReK¯1 is the real part of the complex Clausius–Mossotti (CM) factor. The CM factor is a function of the medium (${\underline{\epsilon }}_{m}$) and the cell (${\underline{\epsilon }}_{p}$) complex permittivities, and to first order is given by ${\underline{K}}^{\left(1\right)}=\left({\underline{\epsilon }}_{p}-{\underline{\epsilon }}_{m}\right)/\left({\underline{\epsilon }}_{p}+2{\underline{\epsilon }}_{m}\right)$. Nonetheless, Equation (6) assumes the dielectric particle is a small dipole when compared to the length scale of the field non-uniformity, thus ignoring the strong field gradients near the electrode edges, and the effect they have on the cell polarization.

In view of the limitations of Equation (6), there are two main approaches for numerically calculating the total average force on a polarizable object in a non-uniform electric field. The first one is multipolar DEP theory, which uses higher-order multipolar moments to compute the average force on a particle. In the steady-state sinusoidal field $E\left(r,t\right)$, the multipolar induced-moment n-th order tensor is given by [47]

(7) $${\underline{p}}^{\left(n\right)}\left(r\right)=\frac{4\pi \epsilon_{m}a^{2n+1}n}{\left(2n-1\right)!!}{\underline{K}}^{\left(n\right)}{\left(\nabla \right)}^{n-1}\underline{E}\left(r\right),$$

where ${\left(\nabla \right)}^{n-1}$ represents n−1 gradient operations, and ${\underline{K}}^{\left(n\right)}$ is the n-th order CM factor or polarization coefficient ${\underline{K}}^{\left(n\right)}=\left({\underline{\epsilon }}_{p}-{\underline{\epsilon }}_{m}\right)/\left[n{\underline{\epsilon }}_{p}+\left(n+1\right){\underline{\epsilon }}_{m}\right]$. Equation (7) can be used to compute the time-averaged electrostatic force on the n-th order multipole at position r, so that by summation of all multipolar contributions, the total average DEP force reads [47]

(8) 〈FDEPr〉=12∑k=1∞Rep¯n⋅n∇nE¯*n!,

where * denotes a complex conjugate operation, and ${\left[\cdot \right]}^{n}$ represents n dot products. The force component counterbalances the hydrodynamic forces, then follows by $F_{\mathrm{DEP}}^{x}=\langle F_{\mathrm{DEP}}\left(r\right)\rangle \cdot \hat{x}$. An advantage of the multipolar approach is that electric field variations can be accounted for as higher-order poles are introduced. The method, however, is not well suited for situations in which the length scale of electric field variations is comparable to the particle size (e.g., when cells are within a distance h=a to the electrode edges). The second method involves performing an integration of the total electrostatic stress on the surface of the particle to compute the total force on the cell. As with the hydrodynamic force, the relevant *x*-component of this force according to Figure 2b is

(9) $$F_{\mathrm{DEP}}^{x}=\oint_{S}dA\hat{n}\cdot \langle M\left(t\right)\rangle \cdot \hat{x},$$

where $\langle M\left(t\right)\rangle$ is the time-averaged Maxwell Stress Tensor (MST), given by [48]

(10) 〈Mt〉=14Reϵ¯mEE¯*+E¯*E¯−E¯2I.

Notice that if $M\left(t\right)$ is obtained with respect to the orientation of the rotated system of coordinates of Figure 2b (inset), then the DEP $y^{\prime }$-component force can alternatively be computed by dot product with $\hat{y^{\prime }}$ in Equation (9), and then transformed via $F_{\mathrm{DEP}}^{x}=F_{\mathrm{DEP}}^{y^{\prime }}\sin\theta$.

Overall, the MST approach to force calculation lends itself to finite element analysis, where the high electric field non-uniformity created by arbitrary electrode configuration and complex geometry defining S can be fully incorporated. For practical purposes, however, a challenge exists in considering the different length scales components (e.g., nano-scale cell membrane thickness versus hundreds of microns for the device height). We have therefore adopted a hybrid approach in which we computed the cell frequency-dependent dielectric properties via the Maxwell Garnett mixing formula. This formula can be used to calculate the effective complex permittivity ${\underline{\epsilon }}_{n}^{\mathrm{eff}}$ of a nucleated particle by progressively simplifying it to a homogeneous dielectric sphere that considers the dielectric properties of its interior components (called inclusions). The equation reads [49,50] as follows:

(11) $${\underline{\epsilon }}_{n}^{\mathrm{eff}}={\underline{\epsilon }}_{n}\left[\frac{1+2\phi_{n-1,n}{\underline{K}}_{n-1,n}}{1-\phi_{n-1,n}{\underline{K}}_{n-1,n}}\right],$$

where $\phi_{n-1,n}$ is the volume fraction of component n−1 (the inclusion) within component n with permittivity ${\underline{\epsilon }}_{n}=\epsilon_{n}\epsilon_{0}-i\sigma_{n}/\omega$, and $\epsilon_{0}$ is the vacuum permittivity. In Equation (11), ${\underline{K}}_{n-1,n}$ is the CM factor of a particle (inclusion n−1) within dielectric media n. In the case of concentric spheres having inner radius r−δ and outer radius r, the volume fraction is ${\left(\frac{r-\delta }{r}\right)}^{3}$. In this way, the intracellular complexity of the cells was accounted for in our calculations, while the spatial non-uniformity of the electric field was modelled via Equations (9) and (10).

## 3. Materials and Methods

### 3.1. Device Fabrication 

The microfluidic channels were fabricated via a multi-step soft lithography technique. First, a negative photoresist SU-8 2015 (Kayaku Advanced Materials, Inc., Westborough, MA, USA) was used to pattern the 20 µm narrow constriction for the hydrodynamic focusing region. Then, to fabricate channels with 250 µm height, a two-step patterning strategy (step 1:100 µm, and step 2:150 µm) was performed using SU-8 2075. Compared to single-step lithography, this strategy minimized uneven channel heights across the device. After the fabrication of the SU-8 mold, Teflon coating was used to avoid adhesion of Poly (dimethylsiloxane) and PDMS (Sylgard 184, Dow Corning, Midland, TX, USA) to the mold. In the next step, the PDMS base and curing agent were mixed at a ratio of 10:1, poured on the mold, degassed for 1 h in a desiccator, cured for 4 h in a 65° oven, and peeled off from the mold. IDA electrode batch fabrication on glass slides was performed using the lift-off technique. For this, the positive photoresist MICROPOSIT^TM^ S1813 was used to pattern the design on the glass slides. Then, 300°A of chromium (Cr) and 1000°A of gold (Au) layers were deposited on the slides using the e-beam evaporation technique. Subsequently, by using sonication in a bath of acetone, the photoresist was removed to obtain the final electrode design. Finally, the PDMS microfluidic chips and glass slides containing IDA electrodes were treated via oxygen plasma, aligned, bonded, and baked for at least 1 h in a 120° oven. 

### 3.2. Experimental Setup and Standard DEP Chip Operation 

The fluids were driven using syringe pumps (Harvard Apparatus, Holliston, MA, USA). Specifically, two pumps were connected to the inlets and operated in infusion mode for sample and sheath flows. Additionally, to have a stable flow in the chip and avoid cell loss at the outlets and tubing, two pumps were used in withdrawal mode at the outlet ports. The z-focusing and sorting IDA electrodes were separately connected to two 33210A waveform/function generators (KEYSIGHT, Santa Rosa, CA, USA). As for z-focusing IDA electrodes, a voltage amplifier (HVA200, Thorlabs, Newton, MA, USA) was used to amplify the signal from the waveform generator. Prior to DEP sorting, the chip was first washed with 70% ethanol at 10 µL/min for 5 min and filtered milliQ (MQ) water at 10 µL/min for 15 min to remove bubbles. In the next step, to avoid unwanted cell adhesion to electrodes and clogging at the shallow constriction at the flow-focusing region, 5% bovine serum albumin (BSA) in filtered MQ water was pumped into the chip at 10 µL/min for 30 min to coat the surface of IDA electrodes and channel walls. The chip was then washed with an ultralow conductive (~85 µS/cm) DEP buffer [51] (CytoRecovery^®^, Blacksburg, VA, USA) at 10 µL/min for 10 min to remove any remaining BSA solution. To perform sorting, the cells were suspended in DEP buffer with a concentration of 1 × 10^6^ cells/ml right before the experiments and introduced into the chip. Furthermore, to maintain cell viability, they were immediately cultured in the cell culture media after collection from the sorter. 

### 3.3. Cell Culture and Viability Test

IMDM supplemented with 10% FBS was used for culturing K562 cells. The cells were grown in a humidified atmosphere of 5% CO_2_/95% air at 37 °C. Calcein AM (ThermoFisher Scientific, Waltham, MA, USA) was used to perform viability tests. For preparing dead K562 cells, the cells were incubated in a 65 °C hot bath for 45 min. 

### 3.4. Numerical Modelling 

Three independent finite element analysis simulations were carried out using COMSOL Multiphysics 5.3 software (COMSOL Inc., Burlington, MA, USA) for (1) hydrodynamic force simulations for force scaling analysis (Supplementary Note S1), (2) dielectrophoretic force simulations for force scaling analysis (Supplementary Note S2), and (3) flow simulation and particle tracking for the coupled hydrodynamic-DEP 3D cell focusing region (Supplementary Note S3). The simulations were performed on a desktop computer with an Intel(R) Core (TM) i7-8700 CPU at 3.20 GHz, 16 GB of RAM, and an NVIDIA GeForce GTX 1050 Ti graphics card. Detailed discussions on the mesh independence study and level of discretization are presented in Supplementary Note S4.

## 4. Results

### 4.1. Scaling of Hydrodynamic and Electromagnetic Forces for High-Throughput DEP Sorting

**Figure 3:**
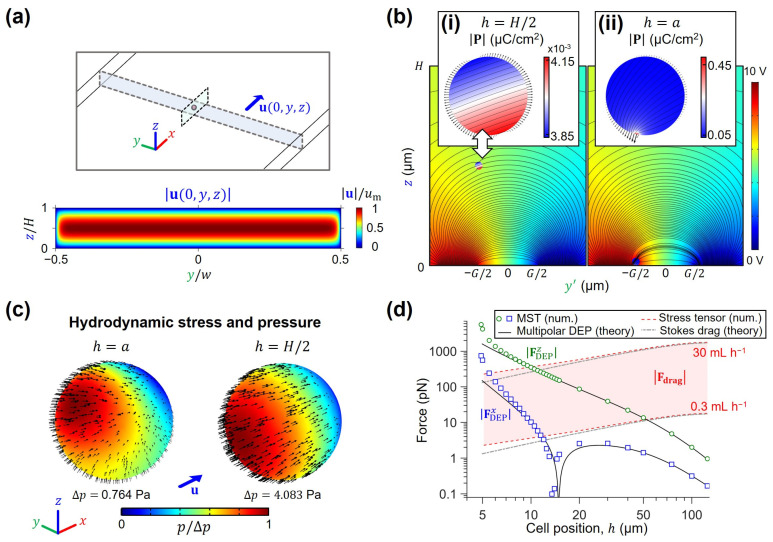
(**a**) Transversal plane view of the rectangular portion of the microfluidic device. Colormap of the flow field magnitude (Equation (4)). (**b**) Electric potential and electric field line distributions with cell present at positions h=a and h=H/2. (**b**(**i**,**ii**)) Polarization density magnitude inside cells, for two different h positions. Lines represent the electric field, and vectors at edges display the MST components used to compute forces. (**c**) Hydrodynamic stress and pressure magnitude on the surface of cells for different h. (**d**) Magnitude of the different forces experienced by K562 cells inside the chip, for different h and flow rate (Q) conditions. For both hydrodynamic and electromagnetic forces, numerical solutions (Equations (2) and (9)) are compared against theoretical models (Equations (5) and (8)).

Simulation of the velocity (Figure 3a) and electric (Figure 3b) fields enables visualization of the stresses generated on the surface of an ideally spherical, stationary cell with radius a. As seen in Figure 3b, proximity to the planar electrodes results in strong electric field non-uniformities, which have an impact on the polarization density distribution inside cells. In Figure 3b(i), we show the calculated polarization density of a cell far from electrodes (h=H/2= 125 µm), which illustrates only a negligible change of ~0.3 × 10^−3^ µC cm^−2^ throughout the volume of the K562 cell. The MST vectors distributed on the surface of the cell demonstrate the closely balanced forces at opposite ends, explaining the negligible DEP forces. In contrast, Figure 3b(ii) illustrates the marked increase in polarization density distribution towards the electrode edge when h~a. In this case, the MST vectors indicate a strong electric force imbalance at the polarized ends of the induced dipole, which cause the net DEP displacement [45]. Interestingly, almost the totality of the polarization change (~0.4 µC cm^−2^) is contained within a small volume inside the cell. This strong polarization not only increases the total DEP force, but also acts as an electric field source, thus contributing to the sharp field non-uniformity. Figure 3c further illustrates the hydrodynamic stress and pressure acting on the surface of the cell as a function of position h. When a cell is close to the bottom of the microchannel (h~a), the asymmetrical shear and pressure experienced by the cell with respect to its center of mass results in unbalanced top and bottom forces, explaining the experimentally observed rolling motion of K562 on top of electrodes. Conversely, a cell located mid-channel results in symmetrically distributed stresses as seen by the colormap plots and arrow fields. 

Using the results from both electromagnetic and hydrodynamic simulations of Figure 3b,c, the drag and DEP forces were plotted as a function of position in Figure 3d. This graph portrays the scaling of both types of forces as calculated by the different methods presented in the Theory section. In all calculations, K562 properties were used (Table S1) by building a simplified multi-shell dielectric model (Figure 2a) using the Maxwell Garnett mixing formula in Equation (11). We thus calculated a force map in Figure 3d where square markers demarcate the $F_{\mathrm{DEP}}^{x}$ magnitude. Here, we see that this force exhibits a sharp decrease in magnitude near h~15 µm due to a sign shift. This change can be interpreted as the x-directed force pointing to the next available electrode as cells are flowing above the planar electrodes. In addition, circle markers are used to show the $F_{\mathrm{DEP}}^{z}$ magnitude, which are indicative of the strength of the electrodes to exert positive DEP attraction on cells. As expected, the z-directed component has no sign changes because force always results in a net movement towards the electrode plane. Moreover, the shaded region in Figure 3d shows the magnitude of fluidic drag balancing the x-directed DEP forces, which span the force range of ~2 pN to 1790 pN for total device cross-sectional flow rates (Q_total_) of 0.3 mL h^−1^ to 30 mL h^−1^ (h~a to h~H/2, respectively). Dielectrophoretic forces are known to have poor scaling on the bulk of the flowing sample, where the effect of planar metal electrodes is negligible [52]. Our numerical simulations and K562 sorting experiments thus confirm that for the channel height of 250 µm and high throughput flow rate targeted in this study (i.e., Q_total_ = Q_sample_ + Q_sheath_ = 30 mL h^−1^, where Q_sheath_ = 2 Q_sample_), it is strictly necessary to have ~10–100 pN DEP forces, which can only be achieved below the $h_{critical}$ ~10 µm mark.

To further illustrate the effect of h on the trajectory that cells take as they traverse the microfluidic channel, one can numerically estimate their traveling time via Runge-Kutta integration of the force balance between Stokes drag and DEP forces. We define traveling time as the time it takes a cell to reach the bottom electrodes via positive DEP attraction, given the starting position (0,0,h). As seen in Figure S4, traveling time closely scales as $\propto h^{3}$ [s m^−3^] in the range 10–100 µm (R^2^ = 0.9712). This timescale can thereby be used to estimate the length of the electrode active region that must be used to bring cells close to electrodes, given an operation flow rate or a desired particle traveling velocity in the flow direction. Furthermore, this analysis demonstrates that h must be in the order of the cell diameter (2a) for traveling time to be sharply decreased. Our microfluidic step has therefore been designed to match this scale, and electric signals were applied intermittently to release cells when adhered to electrodes. 

### 4.2. Optimizing the Operational Parameters for the Hydrodynamic-DEP 3D Cell Focusing Module

**Figure 4:**
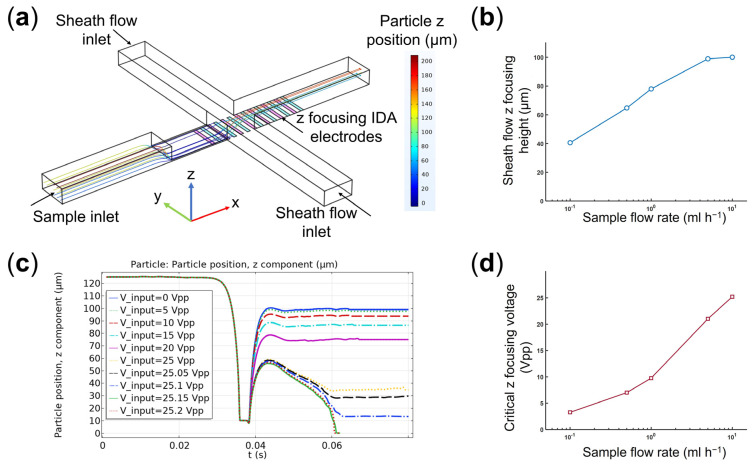
(**a**) The microchannel geometry with the corresponding boundary conditions studied in the numerical modelling, (**b**) the focused high position (z) of cells by 3D hydrodynamic focusing on different sample flow rates. The results are obtained for a sheath flow rate of 20 mL h^−1^ and in the absence of z-focusing IDA electrodes, (**c**) particle trajectory results (z position) to study the performance of the coupled hydrodynamic-DEP 3D cell focusing module at different voltages applied to IDA electrodes, and (**d**) critical z focusing voltages obtained for different sample flow rates to focus cells within ≤10 µm of sorting electrodes. The results are obtained for a sheath flow rate of 20 mL h^−1^.

For each sample flow rate, numerical modelling (i.e., solving for flow field, electric field, and particle tracing) was used to find optimum combinations of sheath flow rate and electric field voltage applied to the IDA electrodes for effective cell focusing (Figure 4a, Video S1). Specifically, based on the force scaling analysis results (Figure 3), the hydrodynamic-DEP 3D cell focusing module should focus the cells to be positioned within less than 10 µm of the sorting electrodes. In the absence of the DEP cell focusing, we first examined the effect of 3D hydrodynamic focusing and found the optimum sheath flow rate (Q_sheath_) of 20 mL h^−1^ to focus the cells at a distance ≤ 100 µm to the bottom of the microchannel for 0.1 mL h^−1^ ≤ Q_sample_ ≤ 10 mL h^−1^ (Figure 4b). According to the results, lower ratios of the sample to sheath flow rates (Q_sample_/Q_sheath_) result in lower z-focusing heights.

In the next step, by fixing the sheath flow rate at 20 mL h^−1^, we evaluated how changes in the voltage applied to the z-focusing IDA electrodes would affect the particles’ final z position. As an example, Figure 4c shows particle tracking analysis for a sample flow rate of 10 mL h^−1^. Based on the results, increasing the voltage reduces the final focused height of the cells by pulling them toward the electrodes via positive DEP (Video S2). Further increase in the applied voltage (e.g., 25.15 V_pp_ and 25.2 V_pp_ in Figure 4c) results in cell trapping to the electrodes. We extended this analysis to find the critical voltage required to achieve the desired cell focusing (z_focusing_ ≤ 10 µm) for the sample flow rates of 0.1 to 10 mL h^−1^ (Figure 4d).

### 4.3. Evaluating the Sorting Performance of the Microfluidic DEP Chip 

**Figure 5:**
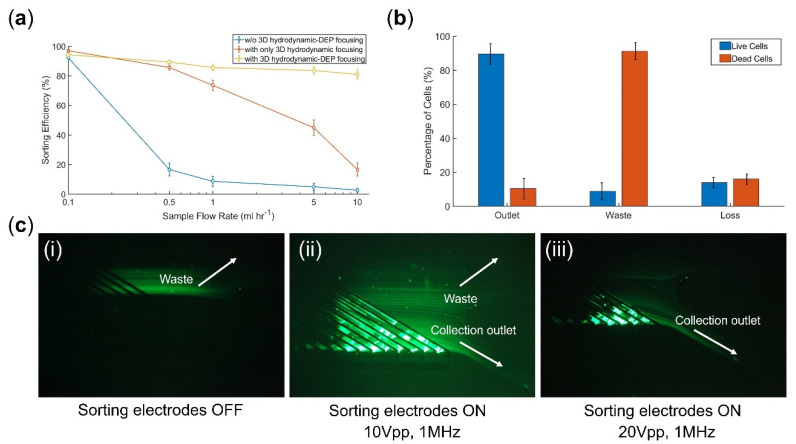
(**a**) Sorting efficiency with respect to sample flow rate for three different DEP sorter chips with and without 3D hydrodynamic and DEP focusing modules. (**b**) Live/dead sorting at 10 mL h^−1^ throughput. (**c**) Snapshots of cell sorting at different stages: (**i**) sorting electrodes OFF: the cells move to the waste in the absence of DEP force, (**ii**,**iii**) Sorting electrodes ON: DEP force deflects the live cells (stained by Calcein AM) to the collection outlet with 20 Vpp and 1 MHz as the optimum parameters. Quantitative data were presented as mean ± standard error (SE). For all experiments, sample size n = 3. The error bars were obtained by three technical replicates.

To better understand the role of the 3D hydrodynamic focusing module and its coupling with the z-focusing IDA electrodes, we first experimentally evaluated sorting performance at different sample flow rates for three different sorter chips: (1) a conventional DEP sorter chip without the coupled hydrodynamic-DEP 3D cell focusing module upstream of the sorting region, (2) a DEP sorter chip with only 3D hydrodynamic focusing module (z-focusing electrodes OFF), (3) a DEP sorter chip with coupled hydrodynamic-DEP 3D cell focusing module. For this purpose, we introduced live K562 cells (cell viability = 93 ± 5%) at the sample inlet. Based on the channel design and flow rate ratios, the cells would initially be guided to Outlet 1 (Figure 1b) with sorting electrodes OFF. Thus, we quantified the sorting performance by calculating the percentage of cells deflected to Outlet 2 (Figure 1b) by the activated sorting electrodes (e.g., 20 Vpp at 1 MHz frequency for 30 mL h^−1^ total flow rate). According to the results (Figure 5a), in the absence of upstream focusing modules, we observed a dramatic decrease in sorting efficiency for sample flow rates above 0.1 mL h^−1^. The integration of a 3D hydrodynamic focusing module into the system circumvents this limitation to some extent for flow rates up to 1 mL h^−1^. However, as numerically investigated (Figure 4b), for flow rates >1 mL h^−1^, the sheath flow alone is unable to effectively focus the cells close to the sorting electrodes. As a result, there is a rapid decrease in sorting efficiency in such operating regimes. In contrast, by incorporating the coupled hydrodynamic-DEP 3D cell focusing module, we could efficiently deflect cells (>85%) to the designated outlet for flow rates up to 10 mL h^−1^ (Video S3) with no significant change in cell viability (<5% change). However, there is still a slight decrease (<10%) in sorting efficiency when increasing the flow rate from 0.1 to 10 mL h^−1^ (statistically significant with *p* < 0.05), which can be improved by further increasing the total channel height (i.e., >250 µm). We also investigated a sorter design with only a DEP z-focusing module (i.e., w/o hydrodynamic focusing) upstream of the DEP sorting region. In this case, the sorting efficiency still dropped below 50% for flow rates >1 mL h^−1^. We found out that the DEP z-focusing module alone could not effectively focus cells within the 250 µm channel height used in this study due to the exponential decay in the electric field. Such a result further demonstrates the necessity of coupling both hydrodynamic and DEP-focusing modules. 

It should be mentioned that the optimum critical z-focusing voltages, obtained via numerical modelling (Figure 4d), were calculated to ensure desired cell focusing for a wide range of initial cell positions along the microchannel height (i.e., 5 µm to 245 µm in this study). Thus, both numerical modelling and experimental results confirmed cell trapping by z-focusing electrodes for the cells closer to the electrodes. As a result, during the experiments, we intermittently turned off the z-focusing electrodes (80 ms ON and 20 ms OFF) to release the trapped cells. Similarly, we adopted such an ON/OFF strategy for the sorting region (1 s ON and 100 ms OFF) to avoid any unwanted cell adhesion to the electrodes (Video S3). 

In the next step, we tested the chip performance for live/dead cell separation at 10 mL h^−1^ sorting throughput. Samples with ~50% cell viability (by proportionally mixing live with dead control groups) were introduced into the chip, and the focusing and sorting electrodes were set at 25.5 Vpp at 1 MHz (80 ms ON and 20 ms OFF) and 20Vpp at 1 MHz (1 s ON and 100 ms OFF), respectively. According to the sorting results (Figure 5b), 89 ± 6% and 91 ± 5% separation purities were achieved for live and dead cells, respectively. In addition, 85 ± 3% live cell recovery (i.e., 15 ± 3% cell loss) was calculated by

(12) $$\%Recovery=100-\%Loss=\frac{number\;of\;target\;cells\;collected\;in\;the\;sorted\;fraction}{original\;number\;of\;cells\;in\;the\;pre-sort\;sample}.$$

## 5. Discussion 

Extensive efforts have been made to develop high-throughput DEP-based cell separation methods (Table S2). DEP field-flow fractionation (DEP-FFF) was among the first techniques capable of achieving high sorting rates up to 4.5 mL min^−1^ [53,54]. However, in contrast to continuous-flow mode, DEP-FFF is typically operated in batch mode due to its reliance on a prior cell levitation step. Furthermore, separating cells that are equilibrated in multiple streams is challenging, as it is sensitive to variations in device depth and its orientation with respect to the gravity force [55]. Yan et al. integrated DEP with hydrophoretic focusing enabled by crescent-shaped grooves and demonstrated high-throughput (~9 mL h^−1^) particles/cells focusing [56]. This approach depends on negative AC DEP to levitate cells and bring them closer to the grooves. Hence, like DEP-FFF, balancing gravity and DEP can be challenging for sorting cells into different streamlines. In addition, for continuous-flow sorting, cells should be initially focused near the electrodes for consistent levitation. In another novel method, termed electrophysiology-activated cell enrichment (EPACE), Faraghat et al. reported high separation rates of more than 320,000 cells s^−1^ [57]. Within their chip, consisting of parallelized arrays of through-holes surrounded by electrodes along the bore, separation works by trapping one type of cell along the electrodes via positive DEP while allowing the other type to pass through via negative DEP. Despite the advantages EPACE has, the trap and release strategy can prove challenging when sorting more than two types of cells as several rounds of device operation are needed. Luo et al. integrated a gravitational sedimentation-based pre-focusing module upstream of their DEP sorting region to achieve sorting throughput of 12.5 µL min^−1^ [58]. Zhang et al. combined inertial microfluidics with DEP and demonstrated tunable separation of 5 µm and 13 µm polystyrene particles at sample flow rates up to 100 µL min^−1^ by using a top sheath flow to focus the cells near electrodes [59]. Pesch et al. proposed a novel system based on open porous microstructures for high-throughput (up to 11 mL min^−1^) DEP filtration [60,61]. However, the design is prone to low cell recovery and the chance of clogging at high cell concentration. Nie et al. developed a DEP sorter using 3D track electrodes made of silver-PDMS (AgPDMS) composite and demonstrated continuous sorting of HeLa cells from lymphocytes at 1.2 mL h^−1^ [62].

In this work, we presented a new approach for high-throughput DEP sorting where the integration of a coupled hydrodynamic-DEP 3D cell focusing module upstream of the sorting region enabled device operation at high flow rates by keeping cells close to electrodes where the DEP force is effective. To obtain optimum operational parameters of the DEP sorter chip, we first developed theoretical and numerical models to better understand the underlying physics and key requirements for effective cell sorting. With respect to the scaling analysis of the hydrodynamic and electromagnetic forces, our DEP-MST numerical simulation results are in excellent agreement with the multipolar theory of DEP force by Jones and Washizu [47] for values above h~a (Figure 3d). Similarly, the calculated drag forces were validated via theoretical evaluation of the Stokes drag force (Equation (5)), which agrees with our numerical model only at distances far from the electrode plane. Furthermore, we validated our numerical model with the experimental work by Su et al. [63] on measuring the dielectric properties of particles using a rectangular microfluidic channel with bottom electrodes for particle mobilization (Supplementary Note S5). Taken together, our results indicate that MST and hydrodynamic stress tensor models are better suited for calculating near-plane forces and trapping/sorting conditions. This has important implications for the design of microfluidic devices that aim to forecast cell dielectric properties under force-balancing conditions [63]. Based on the channel geometry and electrode configuration used in our design, our theoretical analysis predicted that the distance between cells and electrodes should be ≤10 µm ($h_{critical}$ = 10 µm) for effective cell manipulation at 10 mL h^−1^ (=166.7 µL min^−1^) sorting throughput. In the next step, we used the $h_{critical}$ as the input to our developed computational fluid dynamics (CFD) model to optimize flow rate ratios and applied electric field parameters for desired cells focusing upstream the sorting region. It should be noted that the presented theoretical and numerical models are valid only for spherical cells (e.g., blood cells and cancer cells) [25,34]. For non-spherical cells/particles (e.g., bacterial cells, DNA, nanotubes, and nanowires), a correction factor needs to be included in the models to account for such irregularities [64,65,66]. This is typically conducted by considering the cell shape as an ellipsoid where cell/particle orientation also plays a key role in its trajectory [67]. In our future work, we plan to extend our model to predict the DEP behavior of non-spherical cells and experimentally validate it by sorting bacterial cells [68,69,70]. For this, the chip design, especially the narrow constriction at the flow-focusing region and configuration of IDA electrodes (e.g., width and gap), should be optimized. Additionally, for both spherical and non-spherical cells, we plan to include the effect of particle–particle interaction (i.e., cell–cell dipole interactions) in our system using the Monte Carlo simulation [71,72,73,74]. 

Utilizing the optimum operational parameters, we experimentally demonstrated live/dead K562 cell sorting at 10 mL h^−1^ (>150,000 cells min^−1^) throughput and could achieve 90% separation purity with 85% cell recovery and no negative impact on cell viability. To obtain consistent and high separation efficiencies at higher flow rates, future work will involve increasing the channel height in the sorting region to >250 µm. This requires parametric optimization of the upstream 3D focusing module, as outlined in this study. In our design, one key point to consider is the extensional fluid stress that cells experience at the narrow constriction due to rapid changes in the fluid velocity. This can potentially lead to significant cell deformation and stretching, causing cell death [75]. For the highest sample flow rate tested in this study (i.e., 10 mL h^−1^), our numerical studies show that cells experience an average shear stress of 21.11 Pa for less than 5 ms (maximum of 186.15 Pa for less than 100 µs) in the narrow constriction. It has been reported that such values for shear along with the corresponding short exposure times maintain cell viability and function [76,77,78], as confirmed by our viability tests. Another important point to discuss is the use of ultralow-conductivity DEP buffer in this study. It has been shown that prolonged exposure to such buffers causes variable DEP response and reduction in viability [79,80]. In this work, we used a novel ultralow-conductivity DEP buffer with a balanced pH and osmolarity to better mimic the physiological properties of cells [51]. In comparison with conventional DEP buffers, Hyler et al. demonstrated that such a buffer preserves cell viability, DEP response, and downstream growth post-DEP manipulation [51]. 

The presented microfluidic device currently demonstrates cell separation based on DEP forces, which are integrative and dependent on several factors, including cell morphology, dielectric composition, and media dielectric, as well as operational parameters and microfluidic chip geometry. As evinced by our flow cytometry analysis in Figure 5b, our device achieves cell separation by viability, which is related to the membrane integrity of cells. The relationship between the permeated state of a cell membrane and the dielectric properties is a well-established phenomenon [81], and thus distinct populations were efficiently separated. Although our experimental demonstration is limited to live/dead separation, the presented strategy is readily compatible with other sorting modalities of planar electrodes (e.g., to sort cells by electrophysiological properties [30]). This would only have the prerequisite that the experienced DEP forces by the populations be significantly different to achieve successful segregation. Further limitations of the current rendition of our device include the 3D focusing step geometry, which physically limits the cell sizes that can be handled by design (i.e., less than 20 µm). On the other hand, smaller cells (<2 µm), however, might require a redesign in the sorting portion of the chip to allow sufficient traveling time for DEP to push cells into the collection outlet (see for instance Ref. [82], where sorting of 1~2 µm sized particles is demonstrated with a slanted array of electrodes).

Compared to the state-of-the-art DEP methods, our system offers DEP sorting at comparable or higher throughputs (above 100 µL min^−1^), while addressing key challenges such as the complexity of device fabrication, operation, and integration to other microfluidic modules. Furthermore, the tunability of the operational parameters enables the platform to be ideal for applications where the sorting of more than two cell types is needed. For example, based on the electrophysiological properties of cells, the z-focusing IDA electrodes can focus specific cell subpopulations to the bottom of the channel for downstream sorting while keeping others well above the effective sorting region. This further distinguishes our platform from techniques that integrate other types of pre-focusing modules using hydrodynamic or gravitational forces [58,59]. 

## 6. Conclusions

In recent years, there has been a growing interest in the development of technologies for isolating target cells from heterogenous cell populations. In this context, DEP-based microfluidic cell sorting techniques have emerged as powerful tools, offering high sorting efficiency, precision, and specificity. However, one key bottleneck is that most DEP systems cannot sort cells at high throughputs. In this work, we focused on developing a simple and tunable DEP sorter with planar IDA electrodes, and explored the possibility of integrating an upstream coupled hydrodynamic-DEP pre-focusing module to achieve high-throughput cell sorting. We showed that a pre-focusing module allows for a significant increase in the channel height (at least 5-fold) by keeping cells close to the IDA sorting electrodes. This integration helped scaling down hydrodynamic forces to be balanced with DEP forces at high sample flow rates (~10-fold increase compared to conventional approaches). Furthermore, to study the underlying principles of our design and optimize the device’s operational parameters, we developed comprehensive theoretical and numerical models that can be used as guidelines for the research community to develop the next generation of high-throughput DEP-based cell sorters. 

## Data Availability

The data that support the findings of this study are available within this article and Supplementary Materials.

## Supplementary Materials

The following supporting information can be downloaded at: https://www.mdpi.com/article/10.3390/mi14101813/s1, Supplementary Note S1: Hydrodynamic force simulations for force scaling analysis; Supplementary Note S2: Dielectrophoretic force simulations for force scaling analysis; Supplementary Note S3: Flow simulation and particle tracking for the coupled hydrodynamic-DEP 3D cell focusing region; Supplementary Note S4: Mesh study; Supplementary Note S5: Numerical model validation; Figure S1: mesh study for the hydrodynamic and electrostatic field simulations; Figure S2: geometries and meshes used in the computation of hydrodynamic and dielectrophoretic forces; Figure S3: experimental validation of the presented numerical model; Figure S4: K562 cell traveling time in microfluidic chip as a function of starting z-position; Table S1: K562 cell properties used in the Maxwell Garnett formula; Table S2: summary of high-throughput DEP-based cell separation methods; Video S1: a demonstration of the CFD results for tracking cells at the coupled hydrodynamic-DEP 3D cell focusing module; Video S2: predicting the behavior of K562 cells at the coupled hydrodynamic-DEP 3D cell focusing module for sample flow rate of 10 mL h^−1^, sheath flow rate of 20 mL h^−1^, and a sine wave with 25 Vpp at 1 MHz applied to z-focusing electrodes; Video S3: a experimental demonstration of the effect of the coupled hydrodynamic-DEP 3D cell focusing module on K562 cell separation at the DEP sorting region (ON-OFF strategy was used to minimize cell trapping to electrodes). References [53,54,56,57,58,59,60,61,62,63,83] are cited in the Supplementary Materials.
