# Supplementary material for: A High-Throughput Microfluidic Cell Sorter Using a Three-Dimensional Coupled Hydrodynamic-Dielectrophoretic Pre-Focusing Module
Source: Micromachines (Basel). 2023 Sep 22;14(10):1813. doi: 10.3390/mi14101813 (PMC10609158; doi:10.3390/mi14101813)
Supplement: Supplementary file 1 [file micromachines-14-01813-s001.zip › micromachines-2533364-supplementary.pdf]

Supplementary Materials

A high-throughput microfluidic cell sorter using a three-dimensional coupled hydrodynamic-dielectrophoretic pre-focusing module

Mohammad Aghaamoo [1,3], Braulio Cardenas Benitez [1,3], Abraham P. Lee [1,2,3,*]

[1] Department of Biomedical Engineering, University of California Irvine, Irvine, CA 92697, USA

[2] Department of Mechanical & Aerospace Engineering, University of California Irvine, Irvine, CA 92697, USA

[3] Center for Advanced Design & Manufacturing of Integrated Microfluidics (CADMIM), University of California Irvine, Irvine, CA 92697, USA

[*] Correspondence: aplee@uci.edu; Tel.: +1 949-824-9691

The supplementary information includes:

Supplementary Notes S1 to S5

Figures S1 to S4

Tables S1 to S2

Other Supplementary Materials for this manuscript include the following:

Videos S1 to S3

Supplementary Note S1: Hydrodynamic force simulations for force scaling analysis

Finite element analysis simulations were carried out using COMSOL Multiphysics 5.3 software (COMSOL Inc., Burlington, MA, USA) on a desktop computer with an Intel(R) Core (TM) i7-8700 CPU at 3.20 GHz, 16 GB of RAM, and an NVIDIA GeForce GTX 1050 Ti graphics card. The Creeping Flow module was used to model the hydrodynamic force on the surface of a stationary cell positioned at (0,0,h). The built geometry was set up to represent a rectangular section of length L of the complete microfluidic device where the flow was assumed to be fully developed. A pressure gradient boundary condition of Δp = 0.63929 Pa was applied between the $(0, y, z)$ and the (L, y, z) planes (Q = 166.7 μL min^{-1}), for a liquid with viscosity of η = 8.4×10^{-4} Pa·s. The symmetry plane of the problem $(x, 0, z)$ was used to simulate only half of the complete geometry. We used a free tetrahedral mesh with a minimum element size of 0.2 μm, maximum element size of 25 μm, element growth rate of 1.3 and a curvature factor of 0.2, with a resolution of 1 in narrow regions. A sphere of radius a and centered at (0,0, h) was used to represent a K562 cell in the microchannel. The line connecting (0,0,0) and (0,0,$h - a$) was refined with a distribution of 50 elements, and lines defining the spherical particle surface were set to have 100 elements. No-slip boundary conditions were applied to all walls, including the stationary cell's surface, but excluding inlet and outlet planes. The total hydrodynamic force on the cell was calculated by using an intop1(var) operation on the cell surface, where var was the stress tensor on the x-direction (i.e., spf.T_stressx). A parametric sweep with a stationary study was set up to calculate the total force as a function of h, with a total of 10 values (start: a, end: H/2). For each parameter iteration, a stationary step was selected to determine the flow field and the hydrodynamic stress tensor on the surface of the particle. A Generalized Minimal Residual (GMRES) iterative solver was used with a maximum number of iterations of 200. A fully coupled, automatic (Newton) nonlinear solver for computation and the total runtime was 5 h 12 min 49 s.

Supplementary Note S2: Dielectrophoretic force simulations for force scaling analysis

An independent COMSOL Multiphysics 5.3 simulation from the hydrodynamic study was used to compute the position-dependent electromagnetic forces on K562 cells. A frequency domain study was carried out using the Electric Currents module to apply an AC signal of 20 Vpp and a frequency of 1.3 MHz on parallel gold electrodes of width w_e 50 µm, separated by G = 75 µm (Figure 2b). We used a rectangular block of width $4(G + w_e) = 500$ µm, depth of 400 µm, and height of H to represent a fraction of the complete channel geometry. Note that because electrodes are slanted by $\theta = 30°$ with respect to the x-axis of the microchannel (Figure 2b), the width of the simulation block spanning the diagonal $\overline{AB}$ (Figure 2b inset) needs not match the width w of the microchannel to represent the field near electrodes to good approximation. A spherical domain centered at $(0, -\frac{G}{2} + \Delta y', h)$ in the rotated coordinate system of Figure 2b was used to represent the K562 cell. A value of $\Delta y' = 1$ µm was used to sightly separate the cell's bottom from the electric field singularity at $(0, -\frac{G}{2}, 0)$ when $h = 0$. A parametric sweep was carried out to find the effect of h on the electric force. The value h was varied from 0 to $H/2$ (channel half height). The symmetry plane $(0, y', z)$ was used to simulate only half of the complete geometry. We used a free tetrahedral mesh with a minimum element size of 0.01 µm, maximum element size of 15 µm, element growth rate of 1.5 and a curvature factor of 0.6, with a resolution of 0.5 in narrow regions. In addition, a maximum element of 0.2 µm was used inside the spherical domain to further refine the cell volume. The line $\overline{AB}$ and the edges of electrodes were set to have 100 element points, while lines defining the spherical particle surface were set to have 100 elements. Moreover, the line connecting (0,0,0) and $(0,0,h - a)$ was refined with a distribution of 50 elements. The total time-averaged electromagnetic force (the DEP force) on the cell was calculated by using an intop1(var) operation on the cell surface, where var was the stress tensor on the y'-direction (i.e., ec.unTeavy). This value was then multiplied by $\sin\theta$ to obtain the x-directed DEP force component (Figure 2b inset). Additionally, the flow-orthogonal DEP force in the z-direction was computed via integration of ec.unTeavz. A parametric sweep with a frequency domain study was set up to calculate the total force as a function of h, with a total of 29 values (start: a, end: H/2). For each parameter iteration, a frequency domain step was selected to apply an electric field of a given frequency (f = 1.3 MHz), which allowed computation of the average Maxwell tensor on the surface of the particle and computation of the total electric force. A Bi-Conjugate Gradient

Stabilized (BiCGStab) iterative stationary solver with a maximum number of 10000 iterations was used to compute the electric potential and electric field distribution in each case. A fully coupled solver was used with an automatic (Newton) nonlinear method for computation, with a runtime of 21 min 23 s.

For all simulations, a multi-shell Maxwell Garnett model was used to represent the cell complex dielectric properties. All the dielectric properties are summarized in Table S1. The complete complex permittivity properties of $\underline{\epsilon}_n^{\text{eff}}$ were applied to the finite element analysis simulations, as well as the multipolar DEP equations. Media outside of the cell spherical domain were assumed to have the properties of low conductivity DEP buffer, with a conductivity of $\sigma_m = 100$ µS cm^{-1} and $\epsilon_m = 78$.

Variable	Symbol	Value
Cell radius	a	4.85 µm
Nucleus radius	r_n	3.5 µm
Membrane permittivity	ϵ_{cm}	8.72
Membrane conductivity	σ_{cm}	1×10^{-6} S m^{-1}
Cytoplasm permittivity	ϵ_{cp}	70.04
Cytoplasm conductivity	σ_{cp}	0.503 S m^{-1}
Nuclear envelope permittivity	ϵ_{ne}	17.05
Nuclear envelope conductivity	σ_{ne}	9×10^{-4} S m^{-1}
Nucleoplasm permittivity	ϵ_{np}	50.12
Nucleoplasm conductivity	σ_{np}	0.902 S m^{-1}
Lipid membrane thickness	δ_{cm}	10 nm
Nuclear envelope thickness	δ_{ne}	40 nm
Complex membrane permittivity	$\underline{\epsilon}_{cm}$	$\underline{\epsilon}_{cm} = \epsilon_{cm}\epsilon_0 - i\sigma_{cm}/\omega$
Complex cytoplasm permittivity	$\underline{\epsilon}_{cp}$	$\underline{\epsilon}_{cp} = \epsilon_{cp}\epsilon_0 - i\sigma_{cp}/\omega$
Nuclear envelope permittivity	$\underline{\epsilon}_{ne}$	$\underline{\epsilon}_{ne} = \epsilon_{ne}\epsilon_0 - i\sigma_{ne}/\omega$
Nucleoplasm permittivity	$\underline{\epsilon}_{np}$	$\underline{\epsilon}_{np} = \epsilon_{np}\epsilon_0 - i\sigma_{np}/\omega$

Table S1. K562 cell properties used in the Maxwell Garnett formula. All properties were obtained from [1].

Supplementary Note S3: Flow simulation and particle tracking for the coupled hydrodynamic-DEP 3D cell focusing region

An independent COMSOL simulation was carried out to solve the flow field, electric field, and particle tracing. To reduce the complexity of numerical modeling, a simplified 3D version of the microfluidic channel was built and meshed (Figure 4a). The 3D mesh mainly consisted of tetrahedra elements with triangular 2D elements on boundaries and surfaces. The Navier–Stokes and continuity equations were solved to obtain the flow field in the system:

$$\rho\left[\frac{\partial v}{\partial t}+\boldsymbol{v}.\nabla\boldsymbol{v}\right]=-\nabla p+\eta\nabla^2\boldsymbol{v}, \qquad \text{(S1)}$$

$$\nabla.\boldsymbol{v}=0, \qquad \text{(S2)}$$

where ρ, ν, p, and η are the fluid density, velocity, pressure, and viscosity, respectively.

The electric field, **E**, was obtained by solving the following:

$$\nabla\cdot(\mu_m\boldsymbol{E})=\rho_f, \qquad \text{(S3)}$$

$$\nabla\cdot(\sigma\boldsymbol{E})+\frac{\partial\rho_f}{\partial t}=0, \qquad \text{(S4)}$$

$$\boldsymbol{E}=-\nabla\phi \qquad \text{(S5)}$$

where μ_m is the medium permittivity, ρ_f is the free charge density, σ is the medium conductivity, t is the time coordinate, and ϕ is the electric potential. After solving the flow and electric fields, Newton's second law was used to predict the particle trajectory in the flow:

$$\frac{d}{dt}(m_{cell}\boldsymbol{v_{cell}})=\boldsymbol{F_{Drag}}+\boldsymbol{F_{DEP}}, \qquad \text{(S6)}$$

where m_{cell} is the mass of the cell, $\boldsymbol{v_{cell}}$ is the cell velocity, $\boldsymbol{F_{Drag}}$ is the drag force applied on the cell, and $\boldsymbol{F_{DEP}}$ is the dielectrophoretic force applied on the cell.

Two different sets of parametric sweep studies were performed to obtain optimum flow rate ratios for hydrodynamic focusing and optimum electric field voltages applied to z-focusing electrodes. For each parametric study, laminar flow, frequency domain, and time-dependent particle tracing modules were used to predict fluid flow, electric field of a given frequency, and cell trajectory, respectively. For the steady-state laminar flow and time-dependent particle tracing modeling, a

GMRES iterative solver was adopted with a maximum number of iterations of 200 and 10000, respectively. For the frequency domain model, a BiCGStab iterative stationary solver was used, with 10000 as the maximum number of iterations. For each case study, solving the three modules resulted in a total runtime of approximately 5 min.

Supplementary Note S4: Mesh study

To validate the selected meshes for the hydrodynamic and dielectrophoretic force calculation, we performed a mesh study by varying the number of mesh elements in the respective simulations. A K562 cell was simulated and placed at h = 125 µm for both simulations, and the total forces were compared against the force convergence value in pN. This difference is presented as a percent change in Fig. S1 a,b. The study was halted at a mesh refinement level of 994,321 elements for the flow field simulation and 4,198,062 elements for the electric field simulation. At this point, the relative percentage differences were calculated as 0.26% for the flow field simulation and 0.477% for the electric field simulation when compared to the previous iteration, demonstrating convergence and a good trade-off between the number of elements.

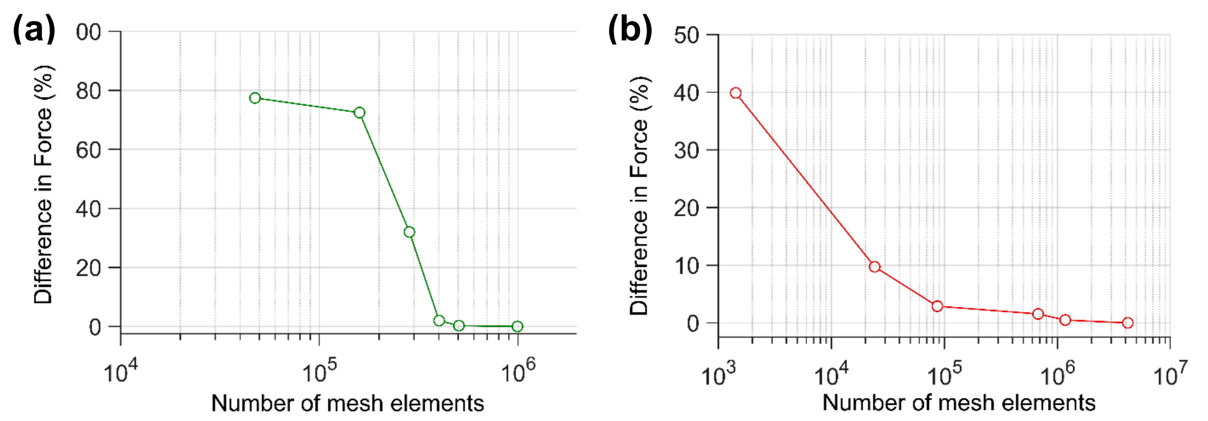

Figure S1. Mesh study for the hydrodynamic and electrostatic field simulations. (a) Percent difference in hydrodynamic force calculation as a function of the number of mesh elements. (b) Percent difference in dielectrophoretic force calculation as a function of the number of mesh elements.

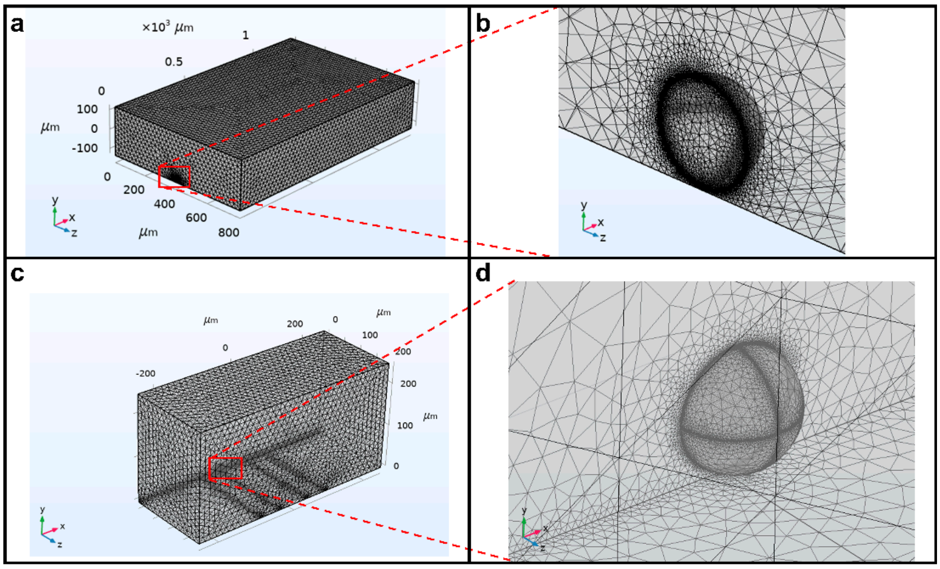

Figure S2. Geometries and meshes used in the computation of hydrodynamic (a,b) and dielectrophoretic forces (c,d). Notice that in the above meshes, only half of the 3D geometry is simulated by exploiting the symmetry of the problem.

Mesh refinement levels of 994,321 elements for the flow field simulation and 4,198,062 elements for the electric field simulation were used. As seen in Fig. S2, the element size is nonlinear in its distribution: element size was reduced as one approaches the cell/particle-liquid spherical boundary. Details on the mapping distributions are provided in Supplementary Notes 1 and 2. It was found that using linear meshing distributions would result in simulations that would not converge to a solution, whereas nonlinear meshes that were dense enough resulted in successful simulations and increasing accuracy. Refer to Fig. 3d in the main manuscript for an assessment of numerical accuracy versus available theoretical models.

Supplementary Note S5: Numerical model validation

To experimentally validate our numerical model, we searched the literature for a study where hydrodynamic and dielectrophoretic forces were involved to control the balance position of particles within a microfluidic chip. Our validation then consisted of predicting said balance positions, given the calculated hydrodynamic and electric stress forces on the surface of a particle. The work of Su et al. details the use of a microfluidic rectangular channel with bottom electrodes used to mobilize particles and measure their dielectric properties [2]. Fig. 3d in that study quantitatively demonstrates the measured particle displacement (or balance position, in µm) due to DEP forces versus the applied voltage at 1 MHz of operation. By implementing all experimental conditions and physical parameters of Table S1 into our DEP-hydrodynamic model, we were able to simulate the predicted particle displacements for the case Q = 2 µl/min. The results of this validation are shown in Fig. S3.

In the case of the applied voltage of 10V, our model predicted a total DEP force of 149.05 pN in magnitude, whereas the DEP force calculated from Ref. [2] for that case corresponded to 147.41 pN, which constitutes a difference of only 1.11%. We therefore conclude that our model reflects the experimental DEP/flow balance to good approximation and the forces calculated from that work. We believe that the combination of these results, along with the theoretical validation presented in Fig. 3d, provides compelling evidence for the practical applicability of our model with a reasonable level of accuracy.

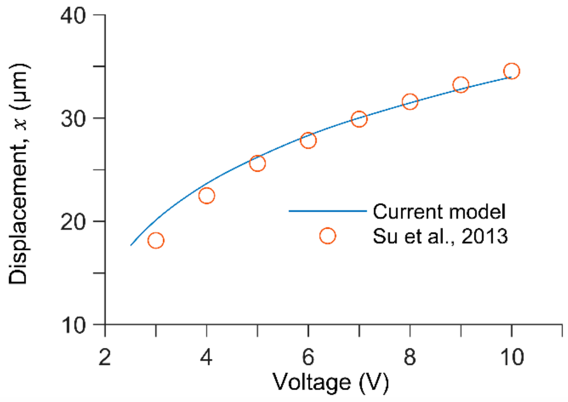

Figure S3. Experimental validation of the presented numerical model, using reported values from Su et al., 2013 [2]. Experimental measurements correspond to the reported displacement of 6 µm particles versus the applied signal voltage. Refer to [2] for further details on the experimental conditions used for measurements.

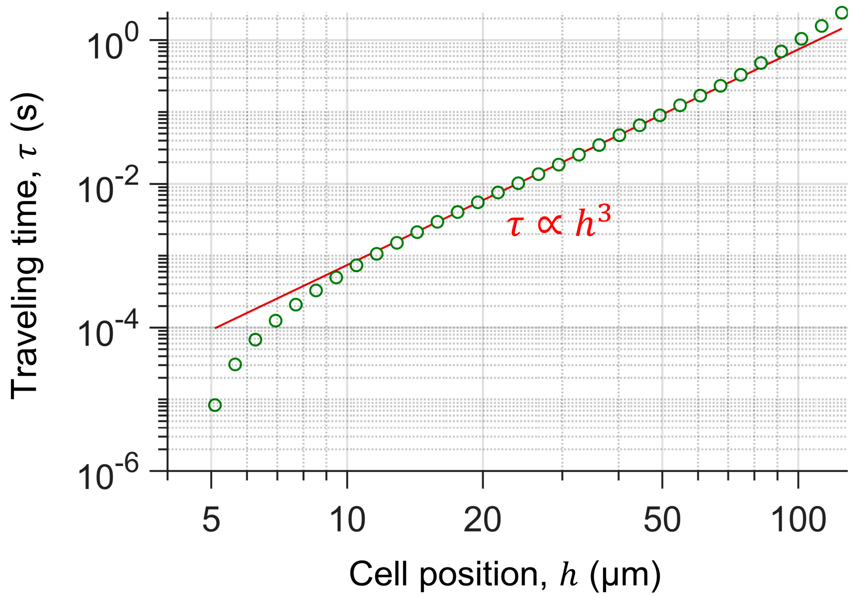

Figure S4. K562 cell traveling time in the microfluidic chip as a function of starting z-position, given by h. A fit to $\tau \sim h^3$ was used to estimate scaling laws for the traveling time.

	Technique	Cell types	Flow rate	Separation purity	Cell recovery
Gascoyne et al. [3,4]	Dielectrophoretic Field-flow-fractionation	Cancer cells (e.g., MDA435, MDA468, and MDA 231) and blood cells (e.g., T-lymphocytes, monocytes, and B-lymphocytes)	Up to 4.5 ml min^{-1}	>90%	10-92%
Yan et al. [5]	Dielectrophoresis-active hydrophoretic focuser	Micro-particles and murine erythroleukemia (MEL) cells	Up to 9 mL hr^{-1}	NA	NA
Faraghat et al. [6]	Electrophysiology-activated cell enrichment (EPACE)	Fibroblasts, Red blood cells, MDA-MB-231 breast cancer cells, and Yeasts	Up to 1 ml min^{-1}	>90%	Up to 96.4%
Luo et al. [7]	Combined gravitational-sedimentation-based prefocusing and dielectrophoretic separation	Human acute monocytic leukemia THP-1 cells, and yeast cells	Up to 12.5 µl min^{-1}	>90%	NA
Zhang et al. [8]	Hybrid dielectrophoresis (DEP)- inertial sorting	5 µm and 13 µm polystyrene particles	Up to 100 µl min^{-1}	38% to 93.8% for 13-µm particles and 62% to 100% for 5-µm particles	NA
Pesch et al. [9,10]	Open porous microstructures	Yeast	Up to 11 mL hr^{-1}	>90%	NA
Nie et al. [11]	Electrode tracks made of conducting-PDMS	HeLa cells, lymphocytes	Up to 1.2 mL hr^{-1}	>90%	80.42%

Table S2. Summary of high-throughput DEP-based cell separation methods.

References

1. Ron, A.; Singh, R.R.; Fishelson, N.; Shur, I.; Socher, R.; Croitoriu, N.; Benayahu, D.; Shacham-Diamand, Y. Dielectric dispersion of suspended cells using 3D reconstructed morphology model. *Bioelectrochemistry* **2009**, *75*, 95-103, doi:https://doi.org/10.1016/j.bioelechem.2009.02.004.
2. Su, H.-W.; Prieto, J.L.; Voldman, J. Rapid dielectrophoretic characterization of single cells using the dielectrophoretic spring. *Lab on a Chip* **2013**, *13*, 4109-4117, doi:10.1039/C3LC50392E.
3. Wang, X.B.; Yang, J.; Huang, Y.; Vykoukal, J.; Becker, F.F.; Gascoyne, P.R. Cell separation by dielectrophoretic field-flow-fractionation. *Anal Chem* **2000**, *72*, 832-839, doi:10.1021/ac990922o.
4. Gascoyne, P.R.C.; Noshari, J.; Anderson, T.J.; Becker, F.F. Isolation of rare cells from cell mixtures by dielectrophoresis. *Electrophoresis* **2009**, *30*, 1388-1398, doi:https://doi.org/10.1002/elps.200800373.
5. Yan, S.; Zhang, J.; Li, M.; Alici, G.; Du, H.; Sluyter, R.; Li, W. On-chip high-throughput manipulation of particles in a dielectrophoresis-active hydrophoretic focuser. *Scientific Reports* **2014**, *4*, 5060, doi:10.1038/srep05060.
6. Faraghat, S.A.; Hoettges, K.F.; Steinbach, M.K.; van der Veen, D.R.; Brackenbury, W.J.; Henslee, E.A.; Labeed, F.H.; Hughes, M.P. High-throughput, low-loss, low-cost, and label-free cell separation using electrophysiology-activated cell enrichment. *Proceedings of the National Academy of Sciences* **2017**, *114*, 4591-4596, doi:doi:10.1073/pnas.1700773114.
7. Luo, T.; Fan, L.; Zeng, Y.; Liu, Y.; Chen, S.; Tan, Q.; Lam, R.H.W.; Sun, D. A simplified sheathless cell separation approach using combined gravitational-sedimentation-based prefocusing and dielectrophoretic separation. *Lab on a Chip* **2018**, *18*, 1521-1532, doi:10.1039/C8LC00173A.
8. Zhang, J.; Yuan, D.; Zhao, Q.; Yan, S.; Tang, S.-Y.; Tan, S.H.; Guo, J.; Xia, H.; Nguyen, N.-T.; Li, W. Tunable particle separation in a hybrid dielectrophoresis (DEP)- inertial microfluidic device. *Sensors and Actuators B: Chemical* **2018**, *267*, 14-25, doi:https://doi.org/10.1016/j.snb.2018.04.020.
9. Pesch, G.R.; Lorenz, M.; Sachdev, S.; Salameh, S.; Du, F.; Baune, M.; Boukany, P.E.; Thöming, J. Bridging the scales in high-throughput dielectrophoretic (bio-)particle separation in porous media. *Scientific Reports* **2018**, *8*, 10480, doi:10.1038/s41598-018-28735-w.
10. Lorenz, M.; Malangré, D.; Du, F.; Baune, M.; Thöming, J.; Pesch, G.R. High-throughput dielectrophoretic filtration of sub-micron and micro particles in macroscopic porous materials. *Analytical and Bioanalytical Chemistry* **2020**, *412*, 3903-3914, doi:10.1007/s00216-020-02557-0.
11. Nie, X.; Luo, Y.; Shen, P.; Han, C.; Yu, D.; Xing, X. High-throughput dielectrophoretic cell sorting assisted by cell sliding on scalable electrode tracks made of conducting-PDMS. *Sensors and Actuators B: Chemical* **2021**, *327*, 128873, doi:https://doi.org/10.1016/j.snb.2020.128873.
